# The price is right!? A meta-regression analysis on willingness to pay for local food

**DOI:** 10.1371/journal.pone.0215847

**Published:** 2019-05-29

**Authors:** Iryna Printezis, Carola Grebitus, Stefan Hirsch

**Affiliations:** 1 Department of Supply Chain Management, W. P. Carey School of Business, Arizona State University, Tempe, Arizona, United States of America; 2 Morrison School of Agribusiness, W. P. Carey School of Business, Arizona State University, Mesa, Arizona, United States of America; 3 School of Management, Technical University of Munich, Munich, Germany; 4 AECP Group, ETH Zurich, Switzerland; University of Florida, UNITED STATES

## Abstract

We study the literature on willingness to pay (WTP) for local food by applying meta-regression analysis to a set of 35 eligible research papers that provide 86 estimates on consumers’ WTP for the attribute “local.” An analysis of the distribution of WTP measures suggests the presence of publication selection bias that favors larger and statistically significant results. The analyzed literature provides evidence for statistically significant differences among consumers’ WTP for various types of product. Moreover, we find that the methodological approach (choice experiments vs. other approaches) and the analyzed country can have a significant influence on the generated WTP for local.

## Introduction

Local food production systems are one of agribusinesses’ major innovations in the last decades [1: 2]. According to Mintel’s Locavore report, consumers in the U.S. are highly motivated to purchase local food, with almost 50% of them stating that they are buying local foods at least on a weekly basis [3]. Moreover, in 2019, Mintel released a report looking specifically at private label food and beverage trends in the US. Testing the priorities for food shopping Mintel asked consumers, which attributes encourage them to buy store brands. Nearly 22% mentioned locally sourced products as a reason [4]. Similarly, in Europe, in 2017, German consumers were asked how often they purchase locally produced foods. Approximately 42% stated ‘very often’, and 45% answered ‘sometimes’ [5]. These examples from across the globe show that local food purchases are a global phenomenon.

**Fig 1:**
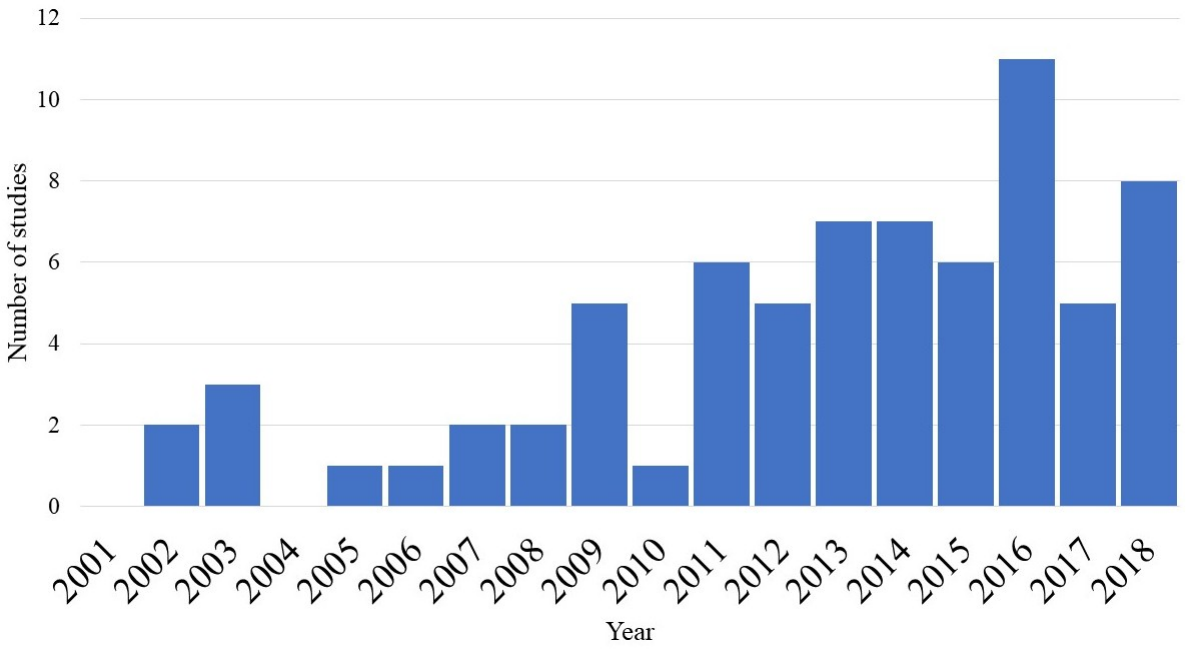
Number of local food WTP studies based on qualitative synthesis.

The shift toward local foods became noticeable in the late 1990s, when consumer behavior studies linked to food purchasing began to show that consumers prefer local food [6, 7]. Since then, the sector experienced significant growth, which triggered extensive empirical research on consumer behavior related to the “local” attribute of the food [2, 8]. A qualitative synthesis, shown in Fig 1 (Please see S1 Table for a list of all articles), reveals the growth in the body of research that investigates consumers’ demand and preferences for local food. Nevertheless, there is still ambiguity regarding consumers’ actual willingness to pay (WTP) for the “local” attribute.

Knowing what consumers are willing to pay for local is important because it provides marketers and policymakers, who seek to promote products as local, with useful information necessary to guide their decisions in developing effective marketing strategies. This, in return, allows growers and producers to capture a proper portion of the consumer surplus as a profit. However, the vast literature on preferences for local food offers heterogeneous information as to what consumers are willing to pay for local. Therefore, the overarching objective of this paper is to provide a concise synthesis of the research results, and to offer a deeper understanding on how labeling food as “local” affects consumers’ WTP. Specifically, taking into account study design and methods utilized to empirically measure WTP, including definitions used and experimental settings employed, we seek to derive a proxy for the value consumers place on the attribute “local”.

The definition of local food usually relates to geographic boundaries. For example, the product has to be produced within 100 miles from the point of sale or within a state border [9] to qualify as “local”. However, there is no consensus regarding the appropriate distance local food can travel before being purchased by consumers. For instance, in the U.S. “local” food is not officially defined. The U.S. Department of Agriculture only states that a product can be considered local if it is transported for less than 400 miles, or within the state in which it was produced [10]. Similarly, in European countries local food is understood in various ways because no uniform definition or standardized label exists [11, 8]. Therefore, German consumers, for example, define local as “region of origin”, which could be a federal state, such as Bavaria, or a certain radius, such as 50 km [11, 12]. As a result, research suggests that consumers’ understanding of the term “local” varies anywhere between state boundaries and a closer proximity, such as “produced within 50 miles” [13, 14, 15, 16, 17]. Regardless of the definition, consumers’ demand and preference for food characterized as *“local”* is experiencing significant growth [18, 19, 20, 21].

Consumers places a high value on the “local” attribute compared to other value-added claims. For example, a study among shoppers in a grocery chain store by Costanigro et al. (2011 [22]) shows that consumers are willing to pay more for both organic and local attributes of fresh Gala apples. However, the WTP for local ($1.18) turns out to be comparatively higher than the one for organic ($0.20). Similarly, conducting face-to-face in-home interviews, Arnoult et al (2007 [23]) find evidence that compared to “certified organic” the “produced locally” claim is valued more for strawberries and lamb chops. Also, interviewing supermarket shoppers in Colorado, Loureiro and Hine (2002 [24]) reveal that consumers are willing to pay more for local, Colorado grown, fresh potatoes compared to food certifications, such as organic and GMO-Free alternatives. In addition, surveying residents in Pennsylvania, James et al. (2009 [25]) show that consumers have a higher WTP for local applesauce compared to organic, low fat, or ‘no sugar added’ applesauce. However, examining the literature, we find that there seems to be a difference among consumers’ WTP for local depending on the experimental settings employed.

Research on local food uses a variety of labels that convey to consumers that a product is locally produced and sourced. This includes but is not limited to: (1) “locally grown” or “local” labels [23, 26, 27, 28]; (2) labels with a local brand name [29]; (3) labels signifying that the product is from a certain state/ region/ city [13, 30, 31, 11]; (4) labels specifying the distance the food has traveled [20, 32, 33]; and (5) labels with a marketing program, such as “Maryland’s Best”, “Jersey Fresh”, “PA Preferred”, “Virginia’s Finest”, “Quality certified Bavaria” [34, 12]. Moreover, the literature on WTP for local food has employed a variety of different study designs, for example, focusing on diverse products, such as, fresh produce [26, 35, 36], animal products [37, 32], and processed food items [38, 12], as well as, regions [17, 32, 39]. In addition, studies on local food utilize different methods to empirically measure WTP, for example, choice experiments [25, 40, 41], contingent valuation [42, 43, 44], and auctions [45, 46, 47]. Given the diversity in methodological characteristics utilized, it is not surprising that there seems to be a variation in reported WTP for “local”.

Previous research shows varying premiums for different labels within the studies, for instance, between ground beef that traveled 100 miles ($1.21) and ground beef that traveled 400 miles ($0) [32]; or blackberry jam that carries labels “Produced in Ohio Valley” ($0.42), “From the region” ($0.25), and State Proud logo ($0) [39]. In addition, previous findings also suggest that there is a difference in premiums for similar labels between studies that use different products: Kentucky-grown Blueberry jam ($2.33) [38]; Minnesota Grown tomatoes ($0.67) [48]; and Arizona Grown carrots ($0.10) [35]. Moreover, research indicates that consumers are willing to pay different premiums for the same products that are characterized by different or similar labels of local, such as, “grown in Ohio” strawberries ($0.45) [15] versus “produced locally” strawberries ($1.55) [23]; “locally grown” apples ($0.22) [26] versus “locally grown” apples ($1.18) [22]; milk from the “region” ($0.29) [12] versus milk “from the local region” ($0.69) [11]; beef from the “local region” ($1.84) [37] versus beef that “traveled 100 miles” ($2.72) [32]. Given the reported differences among premiums, the question remains what the actual WTP for local is. Therefore, the aim of this paper is to assemble all the available evidence on this topic across the literature and determine an estimate of WTP for the “local” attribute.

Given the large number of studies on the demand for local food, we employ a meta-regression analysis (MRA) to determine the WTP for the local attribute. MRA is a popular quantitative technique that allows the researcher to synthesize previous research findings, and to control for the effects of study-specific characteristics, such as analyzed product or applied method, on the resulting empirical estimates of WTP [49, 50]. According to Stanley (2001 [51]) MRA is superior to other meta-analysis approaches, such as qualitative literature reviews, that summarize previous economic research. MRA allows us to analyze the distribution of reported WTP estimates and provides a proxy for the “true” WTP for the “local” attribute under consideration of different product groups. Moreover, MRA is able to evaluate whether publication selection biases, that may emerge due to preference of authors, reviewers and journal editors for statistically significant results, prevail across the underlying literature [49, 52]. Finally, MRA also enables us to determine how methodological and other study-specific characteristics, such as origin and demographic characteristics of recruited participants, affect WTP for the local attribute.

The remainder of the paper is organized as follows. In the next section, we explain the data generation process, and provide a description of the identified variables. This also includes an initial graphical investigation of publication selection bias. In the third section, we present and elaborate on the applied models used to carry out the MRA analysis. The forth section discusses and interprets the results. Finally, the last section offers some conclusions and more general implications, and discusses limitations and suggestions for future research.

## Materials and methods

### Data

When conducting MRA, it is important to follow a clear approach while searching for the relevant literature. Therefore, we follow the “Meta-Analysis of Economics Research Reporting Guidelines” provided by Stanley et al. (2013 [53]). We conduct a thorough review of the scientific literature using the following electronic databases: (i) AgEcon Search, EBSCOhost Electronic Journals Service, JSTOR, ProQuest, PubMed, ScienceDirect, Scopus, Web of Science, Wiley Online Library, and (ii) complementary search in Google Scholar. The search includes studies published in English between January 2000 and June 2018.

To carry out the search, we apply a set of keywords that include “Willingness to Pay”, and “Local Food”, or “Local”, or “Regional”, or “State Grown” to ensure that we identify all relevant literature. We use Boolean strings and combine keywords with operators such as AND, and OR to produce more relevant results. We do not include the word “label” in our keywords as it is not usually specified in the article’s title, abstract or keyword list, but rather is implied in the way the food attribute “local” is communicated to the consumers. Our search examines titles, abstracts and/or article keywords. Even though other definitions of local food, such as, “state grown” or “regional”, are used by the studies, the overarching umbrella term “*local*” is mentioned by the relevant articles in one of these sources.

**Fig 2:**
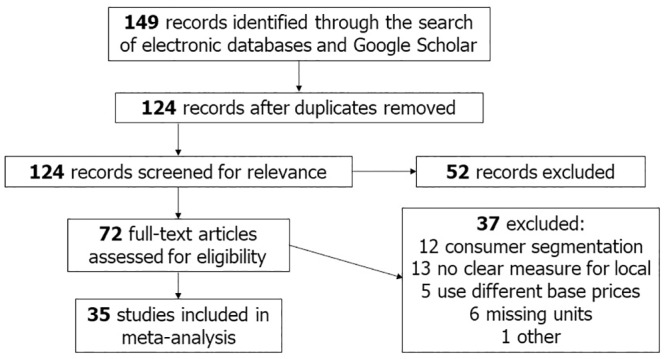
Preferred reporting items for systematic reviews and meta-analyses.

Fig 2 displays the summary of our search results organized using the “Preferred Reporting Items for Systematic Reviews and Meta-Analyses” (PRISMA) template. PRISMA is a popular tool that is used to conduct and report search results [54, 55]. As shown by PRISMA our initial literature identified 149 published papers, including 25 duplicates, such as, same papers yielded by different search engines, earlier versions of papers submitted for conferences, and working papers that were published later. Out of the remaining 124 articles, 52 are not suitable for the present analysis because they do not include a quantitative measure of WTP for “local.” For example, they use qualitative methods to carry out the analysis or do not actually estimate WTP.

Because a uniform interpretation of the analyzed measure is of utmost importance for the feasibility of MRA [56, 57], we assess the remaining 72 studies (see S1 Table for a list of all articles), and identify 35 articles that include a comparable measure(s) of WTP with clearly reported units, such as dollars per pound or dollars per ounce. The remaining 37 studies do not meet the criterion of a uniform interpretation for various reasons. For instance, twelve studies use consumer segmentation techniques or latent class analysis where participants are segmented based on certain variables (e.g., knowledge of organic/local). However, these studies do not provide all information necessary to analyze each consumer segment independently, hence not yielding an individual WTP measure for the local attribute [25, 46, 58, 47]. We also exclude thirteen studies because they do not provide a clear measure for local (e.g., studies that bundle local with other attributes, such as societal benefits or support for local agriculture and environment, or do not explicitly measure WTP) (e.g., [18, 59]). Moreover, we eliminate five studies that use different base prices or only state percentage increase in WTP (e.g., [42, 60]). Another six studies do not provide weight units for the product, and instead state use measurements, such as, “loaf of bread” or “box of cereal” (e.g., [61, 62]). For these studies, it was not possible to derive a comparable WTP measure. Finally, we exclude one study that was a pilot test with only 27 participants in total.

To ensure consistent coding and reduce the likelihood of errors in the data generation process a minimum of two co-authors read each article in the long list. During in-person meetings, the research group resolved disagreements regarding the inclusion of a specific article in our review and clarified other issues, such as, the most appropriate way to code possibly ambiguous findings and methodological categorizations.

Table 1 presents a chronological overview of the 35 articles included in the MRA, providing the year of publication, authors, journal, number of participants, origin (region or country), and type of product analyzed. We also include WTP reported in each article. Multiple WTP estimates for a single product indicate that either more than one sample was utilized (e.g., participants were segmented based on the location of their recruitment), or different definitions of local were applied. Note that in our MRA we focus on a broader set of study design characteristics that potentially drive variation in reported WTP estimates, such as, specific product attributes, demographics of the analyzed sample, definition of local used, and experimental method employed (including hypothetical or non-hypothetical).

**Table 1:** Studies included in meta-regression analysis.

Year	Authors	Journal	Country of research	Total number of participants	Type of product	WTP $/lb
2002	Loureiro, Hine [24]	*Journal of Agricultural and Applied Economics*	US	437	Potatoes	$0.93
2003	Alfnes, Rickertsen [100]	*American Journal of Agricultural Economics*	Norway	106	Beef	$1.06
2006	Stefani et al. [13]	*Food Quality and Preference*	Italy	77	Spelt	$0.17
2007	Arnoult et al. [23]	*International Marketing and International Trade of Quality Food Products*	UK	222	Lamb chops	$1.41
					Strawberries	$1.55
2008	Darby et al.. [15]	*American Journal of Agricultural Economics*	US	530	Strawberries	$0.45; $0.79
2008	Thilmany et al. [18]	*American Journal of Agricultural Economics*	US	1,549	Melons	$0.021
2009	Hu et al. [38]	*Journal of Agricultural and Applied Economics*	US	557	Pure blueberry jam	$2.33
					Blueberry-lime jam	$3.52
					Blueberry yogurt	$0.65
					Blueberry dry muffin mix	$2.51
					Blueberry raisinettes	$6.56
2009	Yue, Tong [48]	*HortScience*	US	343	Tomatoes	$0.67; $0.73
2011	Costanigro et al. [22]	*Agribusiness*	US	300	Apples	$1.18
2011	Nganje et al. [35]	*Agricultural and Resource Economics Review*	US	315	Spinach	$0.18
					Carrots	$0.10
2011	Onozaka, Thilmany-McFadden [26]	*American Journal of Agricultural Economics*	US	1,052	Apples	$0.22
					Tomatoes	$0.38
2012	Hu et al. [17]	*European Review of Agricultural Economics*	US	1,884	Blackberry jam	$0.29; $0.33; $0.41; $0.29; $0.19
2012	Sanjuán et al. [44]	*Appetite*	Spanish regions, French regions	1,219	Beef	$0.42; $0.65; $0.43; $0.40
2013	Grebitus et al. [20]	*Ecological Economics*	Germany	47	Apple	$0.38
					Wine	$0.84
2013	Illichmann, Abdulai [37]	*German Gewisola conference*	Germany	1,182	Apples	$0.14
					Milk	$0.38
					Beef	$1.84
2013	Lopez-Galan et al. 41]	*Spanish Journal of Agricultural Research*	Spain	803	Eggs	$1.36; $0.48
2014	Boys et al. [36]	*Environment*, *Development and Sustainability*	Commonwealth of Dominica	188	Produce	$0.11
2014	Gracia [101]	*Empirical Economics*	Spain	133	Lamb meat	$0.71
2014	deMagistris, Gracia [102]	*International Journal of Consumer Studies*	Spain	171	Untoasted almonds	$4.09
2014	Meas, Hu [30]	*Southern Agricultural Economics Association Annual Meeting*	US	778	Tilapia	$3.83; $5.25
2015	Adalja et al. [32]	*Agricultural and Resource Economics Review*	US	685	Ground beef	$1.21; $0; $2.72; $2.39; $1.47
2015	Bosworth et al. [29]	*Journal of Food Products Marketing*	US	259	Ice cream	$0.20; $0.16
2015	Hasselbach, Roosen [12]	*Journal of Food Products Marketing*	Germany	720	Bread	$0.45; $0.29
					Beer	$0; $0.37
					Milk	$0.24; $0.29
2015	Meas et al. [39]	*American Journal of Agricultural Economics*	US	1,883	Blackberry jam	$0; $0.25; $0.41; $0.27; $0.42
2016	Wägeli et al. [11]	*International Journal of Consumer Studies*	Germany	597	Milk	$0.69; $0.32
					Pork cutlets	$2.77; $2.65
					Eggs	$1.39; $1.08
2016	Dobbs et al. [103]	*Journal of Food Distribution Research*	US	676	Boneless ribeye steak	$5.06
					Ground beef	$1.66
2016	Sackett et al. [91]	*International Journal of Food and Agricultural Economics*	US	1002	Apple	$0.51
					Steak	$2.29
2016	Willis et al. [65]	*Journal of Agricultural and Applied Economics*	US	340	Produce	$0.17
					Animal product	$0.33
2017	Bazzani et al. [104]	*Food Quality and Preference*	Italy	80	Applesauce	$3.40
2017	Mugera et al. [105]	*Journal of Food Products Marketing*	Australia	333	Fruit yogurt	$1.19
					Skinless chicken breast	$1.26
2017	Gumirakiza et al. [106]	*Journal of food products marketing*	US	819	Peaches	$2.30
					Yellow squash	$3.30
					Eggplant	$2.90
2018	Byrd et al. [107]	*Journal of Food Products Marketing*	US	825	Pork chops	$2.04
					Chicken breast	$1.01
2018	Li et al. [108]	*Journal of Agricultural and Applied Economics*	US	1688	Steak Beef	$2.59
					Ground beef	$0.95
2018	Merritt et al. [109]	*Journal of Agricultural and Applied Economics*	US	408	Beef steak	$2.42
					Ground beef	$1.15
2018	Printezis, Gebitus [110]	*Ecological Economics*	US	1046	Tomatoes	$0.80; $0.85

Note: WTP represents the price premium that consumers are willing to pay for the “local” attribute compared to unlabeled origin food.

We then categorize the identified papers in order to derive binary variables for the underlying study design characteristics that might potentially affect the WTP estimates (e.g., [57]). The derivation of these variables has to be conducted under careful consideration of the trade-off between a reasonable number of categories that will adequately capture the variation in WTP observations, and variables with low explanatory power relating to categories that only occur in a few articles (i.e., variables with a high share of 0-observations). The resulting categories are described below and summarized in Table 2.

**Table 2:** Descriptive statistics of WTP meta-data.

Variable	Definition	Mean	Standard deviation	Expected sign
*Dependent variable*				
WTP	WTP for the local attribute in $/lb	1.204	1.325	+
WTP %	WTP for local attribute calculated as % of average product price used by the study	0.292	0.237	
*Precision*				
Number of participants (n)	Number of participants in the study	621.640	539.285	-
*Study design characteristics*				
Year of study	The experiment was conducted after 2011 = 1, 0 otherwise. (If year when the experiment was conducted is not reported it was assumed to be the year of publication [111])	0.372		-
Country of study—US	Experiment was conducted in the U.S. = 1, 0 otherwise	0.605		+
Other countries	Experiment was conducted outside of the U.S. = 1, 0 otherwise	0.395		Base
Animal products	Products used for the study: fish, meat, eggs, or milk = 1, 0 otherwise	0.430		+
Produce	Products used for the study: fruits, vegetables or nuts = 1, 0 otherwise	0.267		Base
Processed products	Products used for the study: food items that underwent processing = 1, 0 otherwise	0.302		+
Local def.–state grown	“Local” was defined as grown or produced within the state = 1, 0 otherwise	0.244		-
Local def.–marketing program	“Local” was defined using a state/region logo/label = 1, 0 otherwise	0.255		Base
Local def.–specific region	“Local” was defined as grown or produced in a specific province, or region = 1, 0 otherwise	0.360		+
Local def.–general	“Local” was defined as locally grown or produced = 1, 0 otherwise	0.140		-
Method—choice experiment	Experiment was carried out using choice experiment method = 1, 0 otherwise	0.860		+
Method—other	Experiment was carried out using other methods, such as auctions and contingent valuation methods = 1, 0 otherwise	0.140		Base
Hypothetical experiment	Experimental study was hypothetical = 1, 0 otherwise	0.802		+
Non-hypothetical experiment	Experimental study was non-hypothetical = 1, 0 otherwise	0.198		Base
Participants`origin—shoppers	Participants recruited at the shopping locations = 1, 0 otherwise	0.477		+
Participants`origin—other	Participants recruited at random or through marketing companies = 1, 0 otherwise	0.523		Base
Number of attributes	Number of attributes used to describe the product	4.128	1.445	-
Age	Average age of the study participants	46.826	4.340	+
Gender	Percent of female participants in the study	59.682	10.121	-
Income	Average income of the study participants in $	50,336.880	18,311.060	+

#### Dependent variable

In our MRA we use the WTP estimates reported by the 35 articles as the dependent variable. We converted the WTP values to $/lb in order to keep the currency across studies consistent. In addition, we define a WTP measure in terms of an extra percentage that consumers are willing to pay over the base price. This was calculated as the average price used by the respective study following, e.g., Dolgopolova and Teuber (2017 [63]). We use percentage values as a robustness check. Also, it is important to point out that the final number of WTP measures identified (final number = 86) is larger than the number of studies included in the MRA (number included = 35), since some of the papers report multiple WTP estimates due to multiple products per study, multiple samples, and/or multiple definitions of local used in a single article.

It can be observed that the mean WTP for the local attribute across the included studies is 1.204$/lb (0.292 when calculated as a percentage premium). Moreover, the mean number of participants based on which the WTP estimate has been generated is reported. This number is an indicator of how precisely the measure of WTP is estimated [52]. As will become apparent below, the relationship between reported WTP estimates and their precision can be an important indicator for the presence of publication selection bias [52: 64]. Table 2 indicates that WTP estimates are, on average, generated based on samples with 622 participants.

#### Independent variables

*Year of study*. Concerning underlying study design characteristics, we first identify the year in which the data was collected. This allows to test if there is a trend over time in WTP for local estimates. For example, as local food gains more popularity, the WTP might have increased over time. On the other hand, as local food becomes more mainstream as time passes, WTP may subside. We find that the included studies focus on average on 2009 data. However, using this *Year of study* variable caused multicollinearity problems in the MRA. Therefore, we use a dummy variable with value one for studies conducted post 2011, and zero otherwise [63]. We choose the year 2011 as a threshold because it constitutes the median year of study across the included literature.

*Country of study*. Additionally, we include a dummy variable, *Country of study-US*, which captures whether the study was conducted in the US. The “non-US” category includes studies conducted in European countries, such as, Germany, Spain and Italy, and one study conducted in The Commonwealth of Dominica. This country specific variable allows us to identify if the reported WTP differs between the US and other countries.

*Type of product*. Literature on WTP for local considers various product types, since consumer preference for local food does not only pertain to fresh products but extends to processed and animal products. For example, surveying randomly selected South Carolina households, Willis et al. (2013 [65]) find that households have a higher WTP for locally grown produce relative to non-locally grown. Conducting an in-store survey among residents of Kentucky, Hu et al. (2009 [38]) find that consumers had a higher WTP for pure blueberry jam, blueberry-lime jam, blueberry yogurt, blueberry dry muffin mix, and blueberry raisinettes with a Kentucky-grown label. Likewise, interviewing consumers in Germany, Wägeli et al. (2016 [11]) find that consumers are willing to pay more for fresh milk, pork cutlets and eggs from the local region. The reported WTP, however, may vary significantly among the products analyzed. Therefore, we separate the included studies by product type leading to three categories: (i) animal products, such as, meat, fish, poultry, eggs, and dairy products, (ii) produce, such as, apples, beans, melons, potatoes, strawberries, tomatoes, yams, and (iii) processed food products, such as, blueberry fruit rollups, blueberry raisinettes, jam, and applesauce. The premium for processed local food can be higher than for unprocessed local food because consumers are willing to pay a higher price premium for value-added shelf-stable products [66]. Contrary to this, there might be a discount for processed local food because consumers may only hold trust towards unprocessed foods, i.e., whole products that were produced in the region, something that is more difficult to evaluate for processed foods considering that multiple ingredients are involved.

*Definition of local*. With regards to the definition of “local”, the literature has focused particularly on the following four categories: (i) State Grown; (ii) marketing program logos and labels: local brand; Grown Fresh with Care in Delaware, Maryland’s Best, Jersey Fresh, PA Preferred, Virginia’s Finest, Quality certified Bavaria; (iii) more precise definitions: product of city, province, or region; (iv) any of the following general definitions of local/ produced locally/ locally grown/ grown “nearby”. The reported WTP might differ across these categories, as consumers seem to prefer a more precise definition of local, such as “produced within 50 miles” [14], or narrower defined geographical boundaries, such as, sub-state regions [13, 17, 30].

*Method*. Several methods have been employed across the literature to carry out the analysis with a majority of research based on choice experiments. As can be observed from Table 2, about 85% of identified WTP estimates have been generated using this method. Moreover, another set of studies has focused on a heterogeneous set of methods, such as, auctions and contingent valuations leading to two main method categories: (i) choice experiment, and (ii) other than choice experiment. This allows us to determine if the WTP estimates yielded by conducting choice experiments differ in WTP from the other experimental methods. Also, we include a dummy variable that captures whether the experiment was hypothetical. Table 2 reveals that 80% of included WTP estimates stem from hypothetical experiments. Since hypothetical studies are often criticized for not being incentive compatible, we examine whether there is a difference in reported WTP. However, since previous studies have demonstrated that hypothetical settings can provide a bias free estimate of marginal WTP [67, 68, 69, 70], we expect to find no difference between WTP estimates generated based on hypothetical and non-hypothetical experiments.

*Participants’ origin*. Moreover, for each study we determine the place participants were recruited from. This leads to two separate categories: (i) store shoppers, i.e., participants surveyed in person at the place of purchase, and (ii) “other” participants that were recruited through a market research company/online-survey database or through random representative samples (e.g., recruited by mail, phone, or at areas with heavy traffic, such as downtown museums or holiday parades). The WTP reported in the studies that used store shoppers as participants might be lower because those participants are in the shopping environment and, thus, more price conscious.

*Number of attributes*. We also include the number of additional attributes the study used to describe the product, since this might have an effect on the resulting WTP estimate. Consumers’ choice satisfaction tends to decrease when complexity of the offered alternatives increases [71, 72]. For example, having alternatives that are described using many different attributes has a significant effect on the ability to make a choice [73, 74]. One reason for this is an intensity of the cognitive effort necessary to make a choice [75]. Therefore, as the number of attributes increases, the complexity of the experiment might have a negative effect on participants’ WTP. On the other hand, if the attribute “local” is more salient among others, the number of attributes used will not affect WTP for local.

*Demographics*. Finally, we account for demographic characteristics of the surveyed participants that may have an effect on the resulting WTP estimate. Among those are the age (the mean reported by a study), gender (% of females), and income [24, 23, 42, 37].

### Graphical analysis of publication selection bias

Publication selection bias refers to a tendency of having a greater preference for estimation and publishing statistically significant results compared to results that do not reveal statistical significance [57]. Stanley (2005; 2008 [52, 76]) shows that the relationship between analyzed estimates and their precision (e.g., standard errors or sample size) can serve as an indicator for publication selection bias. For example, average *t*-statistics around two, which refer to statistical significance approximately at the 5%-level, across the literature of interest are a strong indication for publication selection bias [57].

We use a scatter diagram of the relationship between estimated effects and their precision, also known as funnel plot. This plot can be used as an initial informal indicator for publication selection bias [52, 77]. While the most precise estimates at the top of this plot should be close to the true effect, the less precise ones at the bottom of the plot are more dispersed resembling an inverted funnel shape. Without publication selection bias, estimated effects should vary randomly and symmetrically around the true WTP effect as all imprecise estimates at the bottom of the plot have the same chance of being reported [78]. In turn, if the plot is over-weighted on either one of the sides, this is considered an indicator for publication selection bias [52].

There are several ways to measure precision of estimated effects, with the most common one being the inverse of the standard error (e.g., [52, 56, 79, 57]). However, for the WTP effects analyzed in the present case, standard errors are not available as these effects are calculated as a combination of regression coefficients, for which the calculation of standard errors is not possible without additional information on the underlying estimation. Nevertheless, the sample size (*n*) and particularly its square root (*sqrt(n)*) can also serve as an adequate precision measure because it is proportional to the inverse of the standard error [52, 64]. For example, Stanley (2005 [52]) finds that correlations between (1/SE) and *sqrt(n)* exceed 0.9. Moreover, Sterne et al. (2000 [80]) and Macaskill et al. (2001 [81]) show that in MRA *sqrt(n)* is a superior measure of precision compared to standard errors. While standard errors are estimated values that are affected by random sampling errors, this does not apply to *sqrt(n)* [52]. This is also important for our MRA, as we include precision as an independent variable to explain the variance in reported WTP estimates. Since (1/SE) is measured with error, its inclusion as independent variable in a regression analysis will lead to errors-in-variables bias. In contrast, *sqrt(n)*, although highly correlated with (1/SE), is free of estimation error [52]. Note that focusing on differences between published and working papers to identify potential publication bias is based on the assumption that working papers remain unpublished due to undesirable (i.e., insignificant results). However, it can well be the case that articles are not published and remain working papers due to quality issues with respect to method applied, writing style, etc. These articles do not necessarily have less significant (i.e., less desirable) results. Moreover, rational authors likely already follow a strategy of producing “desirable” results in the initial stages of research while preparing for journal publication. Therefore, including working papers in the MRA in some instances might not help to detect publication bias, while the inclusion of precision as an independent variable is more accurate (e.g. [82, 83]).

**Fig 3:**
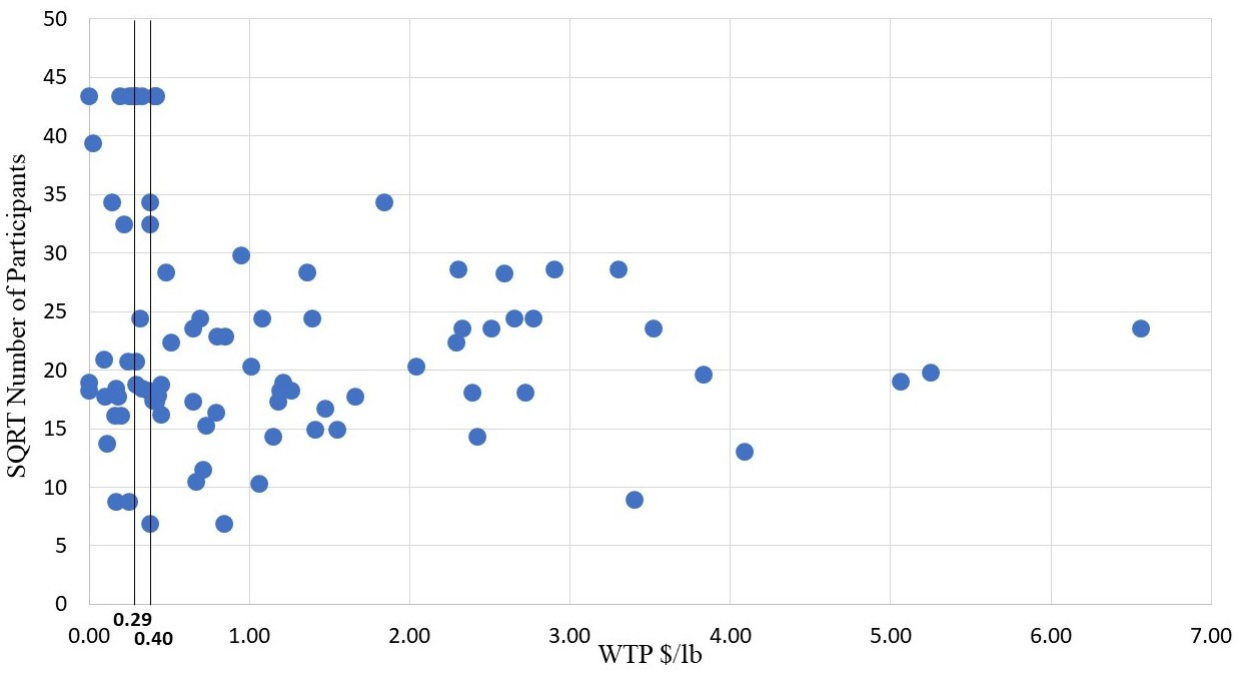
Funnel plot for WTP ($/lb) for local food estimates. Note: “True” values indicated by vertical lines are generated by averaging the 10% and 20% most precisely estimated WTP effects.

Fig 3 displays the funnel plot for the WTP for local food literature. We use the mean of the 10% most precisely estimated WTP effects (measured by *n*) as the measure for the “true” WTP for local food. We do so because as *n* increases standard errors will become smaller, implying that reported WTP approaches the “true” value if *n* → ∞ [52]. For the WTP for local food literature this value is 0.29, represented by the vertical line in the upper panel of Fig 3. As a robustness check we have also calculated the proxy for the “true” WTP by using the 20% most precisely estimated WTP effects leading to a value of 0.40 (lower panel of Fig 3). It can be observed that in both cases the plot is strongly skewed to the right hand side of the “true” value, indicating publication selection bias towards larger WTP estimates.

Despite the usefulness of funnel plots to provide an initial indication of publication selection bias, their weakness lies in the assumption of a single underlying “true” effect. However, different countries, participant types, or product types may be characterized by their own distinct “true” effects [52,57]. The following meta-regression, therefore, provides a more objective test for the asymmetry of WTP estimates that also considers underlying study design characteristics as potential determinants for variation in WTP estimates.

### Meta-regression models

We use MRA to assess previous research quantitatively because MRA is a powerful tool that provides information about relationships of interest by combining results from various previous studies [49]. When summarizing empirical findings of studies, focusing on a similar economic phenomenon, MRA is able to go beyond the estimates that are obtained from individual samples [50]. More precisely, MRA uses differences across studies as explanatory variables in a regression model to explain the effect of interest [84]. When combining information from independent but similar research, meta-analysis “borrows power” from multiple studies to improve parameter estimates that are obtained from a single study [85]. This allows us to estimate a proxy for the ‘true’ WTP effect.

The basic hypothesis of our MRA is that the variation in reported WTP estimates can be explained by the study design characteristics summarized in Table 2. Those include the year of study, number of participants and their origin, product type, definition of “local”, methodological approach, the number of additional product attributes used in the study, and some key demographics of participants. Therefore, we estimate several specifications of the following MRA model (e.g. [52, 76]):

$${WTP}_{i}\;=\;\beta_{0}+\beta_{1}{precision}_{i}+\sum_{k\;=\;1}^{K}\beta_{k}X_{ki}+\varepsilon_{i}$$ (1)

where *WTP*_*i*_ is the dependent variable which captures the *i* = 1, …, 86 identified WTP estimates is regressed on precision, *X*_*k*_ is a vector containing *k* variables related to the study design used to estimate the WTP for “local”, and *ε*_*i*_ is a classical *i*.*i*.*d*. error term.

We start with a simple version of (1) that only includes precision measured by *sqrt(n)* as independent variable:

$${WTP}_{i}\;=\;\beta_{0}+\beta_{1}{sqrt(n)}_{i}+\varepsilon_{i}$$ (2)

The estimated constant of (2) (${\hat{\beta }}_{0}$) provides a proxy for the “true” WTP effect. Without publication selection bias, the observed WTP effects should vary randomly around this “true” effect, independently of their precision (*sqrt(n)*) [52, 64]. Hence, the *t*-test for ${\hat{\beta }}_{1}$, also known as the funnel asymmetry test (FAT), can be used to detect publication bias. Accordingly, rejection of H_0_:$\;{\hat{\beta }}_{1}\;=\;0$ indicates publication bias [52]. The test of H_0_: ${\hat{\beta }}_{0}\;=\;0$, also known as precision effect test (PET), serves as an indicator for the presence of a significant WTP for “local” effect after correction for publication bias [52]. As a robustness check, we also estimate (Eq 2) using the number of participants (*n*) as the precision measure.

Our final MRA model extends (2) by additionally considering all variables that capture the study design *X*_*k*_ used to estimate the WTP effect:

$${WTP}_{i}\;=\;\beta_{0}+\beta_{1}{sqrt(n)}_{i}+\sum_{k\;=\;1}^{K}\beta_{k}X_{li}+\varepsilon_{i}$$ (3)

The econometric estimation of the MRA models specified by (2) and (3) involves two hurdles. First, heterogeneous variances used in WTP estimation might lead to potential heteroscedasticity in the error terms (*ε*_*i*_),which causes biased standard errors of (2) and (3) (e.g., [52, 50]). Nevertheless, the square root of the number of participants (*sqrt(n)*) is a good indicator for this heteroscedasticity because it is positively related to the estimation precision [52]. Therefore, to generate efficient estimates of (2) and (3) with corrected standard errors, we use weighted least squares (WLS) regression with *sqrt(n)* as weights [86, 52, 56].

A second hurdle evolves from the fact that the 86 collected WTP effects constitute 35 clusters of estimates from the same study. Consequently, intra-cluster error correlations may affect WTP observations, which would result in biased standard error estimates of (2) and (3) [50, 57]. Therefore, when estimating (2) and (3), we apply several approaches to mitigate intra-study error dependency: (i) WLS with heteroscedasticity robust standard errors; (ii) WLS with cluster robust standard errors; and (iii) wild bootstrapped standard errors. WLS with robust standard errors is considered as the base specification, while WLS with cluster robust standard errors is usually considered as the superior approach, to capture the heteroscedasticity in meta-regression data [77]. Nevertheless, Angrist and Pischke (2008 [87]) show that the minimum number of clusters for its application should be 42. As our data only consists of 35 study clusters, we also apply the wild bootstrap specification as a robustness check, which is particularly suited to meta-regression data with a small number of clusters [88].

## Results

We present our meta-regression results in Tables 3 and 4. As described above, we use *sqrt(n)* and *n* as precision measures and apply three different approaches to correct for intra-study error correlations. Table 3 presents WLS results for the simple model without additional study design covariates (Eq 2). Columns (3) and (4) display the results for the main specification, WLS with cluster robust standard errors. The significant and negative coefficients of *sqrt(n)* and *n* confirm the presence of publication bias already detected by the funnel plot. This finding is consistent across the remaining methods used to control for intra-study error dependence (robust standard errors in columns (1) and (2) as well as Wild bootstrapped standard errors in columns (5) and (6)).

**Table 3:** PET and FAT analysis (using WTP in $/lb).

	WLS with robust SEs				WLS with cluster robust SEs				Wild bootstrap cluster robust SEs			
	(1)		(2)		(3)		(4)		(5)		(6)	
	Coeff.	CI	Coeff.	CI	Coeff.	CI	Coeff.	CI	Coeff.	CI	Coeff.	CI
Constant	2.076*** (0.313)	1.454; 2.698	1.696*** (0.222)	1.253; 2.139	2.076*** (0.454)	1.154; 2.999	1.696*** (0.322)	1.042; 2.350	2.076*** [0.000]	1.225; 2.945	1.696*** [0.000]	1.094; 2.295
sqrt(n)	-0.035*** (0.008)	-0.051; -0.019			-0.035*** (0.012)	-0.060; -0.011			-0.035** [0.020]	-0.060; -0.012		
n			-0.001*** (0.000)	-0.001; -0.000			-0.001*** (0.000)	-0.001; -0.000			-0.001*** [0.002]	-0.001; -0.000
obs	86		86		86		86		86		86	
F	18.610		27.120		8.470		13.300					
Prob >F	0.000		0.000		0.006		0.001					
R^2^	0.083		0.108		0.083		0.108		0.083		0.108	
Adj, R^2^	0.072		0.097		0.072		0.097		0.072		0.097	

Note: Dependent variable is WTP for local; Standard errors in parentheses; p-values in brackets. CI refers to 95% confidence interval

***, **, * indicate significance at the 1%, 5%, and 10%-level, respectively. *sqrt(n)* is used as weight.

**Table 4:** Meta regression results (using WTP in $/lb).

	WLS with robust SEs				WLS with cluster robust SEs				Wild bootstrap cluster robust SEs			
	(1)		(2)		(3)		(4)		(5)		(6)	
	Coeff.	CI	Coeff.	CI	Coeff.	CI	Coeff.	CI	Coeff.	CI	Coeff.	CI
Constant	6.179*** (2.303)	1.563; 10.796	5.173** (2.203)	0.757; 9.590	6.179*** (2.220)	1.616; 10.743	5.173** (2.025)	1.012; 9.335	6.179* [0.056]	2.661; 9.894	5.173* [0.074]	1.903; 8.350
sqrt(n)	-0.065** (0.027)	-0.119; -0.011			-0.065** (0.031)	-0.130; -0.000			-0.065* (0.080)	-0.120; -0.011		
n			-0.002*** (0.000)	-0.003; -0.001			-0.002*** (0.001)	-0.003; -0.000			-0.002*** [0.002]	-0.003; -0.001
Year of study	-0.633* (0.330)	-1.294; 0.028	-0.814** (0.330)	-1.476; -0.152	-0.633* (0.359)	-1.371; 0.106	-0.814** (0.345)	-1.524; -0.104	-0.633* [0.084]	-1.213; -0.025	-0.814** [0.030]	-1.371; -0.243
Country of study—US	0.805** (0.352)	0.099; 1.511	0.785** (0.334)	0.115; 1.454	0.805** (0.386)	0.011; 1.599	0.785** (0.353)	0.058; 1.511	0.805 [0.146]	0.189; 1.487	0.785* [0.088]	0.225; 1.397
Animal products	1.056*** (0.358)	0.339; 1.774	1.025*** (0.352)	0.318; 1.732	1.056*** (0.402)	0.230; 1.883	1.025** (0.402)	0.198; 1.852	1.056** [0.028]	0.402; 1.777	1.025** [0.026]	0.361; 1.734
Processed products	1.415*** (0.534)	0.344; 2.485	1.547*** (0.538)	0.468; 2.625	1.415** (0.575)	0.233; 2.596	1.547*** (0.563)	0.389;2.704	1.415* [0.076]	0.453; 2.359	1.547* [0.052]	0.623; 2.505
Local def.–state grown	0.095 (0.403)	-0.713; 0.902	0.068 (0.394)	-0.723; 0.859	0.095 (0.494)	-0.921; 1.111	0.068 (0.475)	-0.907; 1.044	0.095 [0.876]	-0.713; 0.912	0.068 [0.918]	-0.712 0.841
Local def.–specific region	-0.042 (0.245)	-0.554; 0.450	0.012 (0.230)	-0.449; 0.472	-0.042 (0.229)	-0.513; 0.430	0.012 (0.215)	-0.429; 0.453	-0.042 [0.906]	-0.426; 0.345	0.012 [0.924]	-0.361; 0.378
Local def.–general	-0.079 (0.481)	-1.043; 0.886	-0.238 (0.488)	-1.216; 0.740	-0.079 (0.609)	-1.330; 1.173	-0.238 (0.615)	-1.501; 1.026	-0.079 [0.978]	-1.096; 0.963	-0.238 [0.798]	-1.278; 0.789
Method—choice experiment	2.139*** (0.733)	0.669; 3.608	2.007*** (0.727)	0.551; -3.464	2.139*** (0.764)	0.569; 3.709	2.007*** (0.737)	0.493; 3.522	2.139** [0.060]	0.925; 3.446	2.007* [0.064]	0.854; 3.246
Hypothetical experiment	-0.344 (0.434)	-1.215; 0.527	-0.443 (0.421)	-1.287; 0.401	-0.344 (0.463)	-1.295; 0.607	-0.443 (0.430)	-1.326; 0.440	-0.344 [0.484]	-1.113; 0.474	-0.443 [0.308]	-1.122; 0.316
Participants’ origin—shoppers	-0.334 (0.374)	-1.084; 0.417	-0.583 (0.383)	-1.351; 0.185	-0.334 (0.414)	-1.185; 0.517	-0.583 (0.410)	-1.427; 0.260	-0.334 [0.538]	-1.025; 0.338	-0.583 [0.246]	-1.291; 0.079
Number of attributes	-0.257 (0.188)	-0.634; 0.120	-0.131 (0.173)	-0.477; 0.215	-0.257 (0.196)	-0.659; 0.145	-0.131 (0.181)	-0.502; 0.240	-0.257 [0.308]	-0.598; 0.071	-0.131 [0.532]	-0.452; 0.179
Age	-0.064 (0.040)	-0.144; 0.015	-0.052 (0.039)	-0.130; 0.026	-0.064 (0.041)	-0.149; 0.020	-0.052 (0.040)	-0.133; 0.030	-0.064 [0.296]	-0.136; 0.006	-0.052 [0.338]	-0.120; 0.017
Gender	-0.035* (0.019)	-0.072; 0.024	-0.038** (0.018)	-0.074; 0.002	-0.035* (0.021)	-0.077; 0.008	-0.038** (0.019)	-0.077; 0.001	-0.035 [0.182]	-0.070; 0.000	-0.038* [0.010]	-0.071; -0.006
obs	69		69		69		69		69		69	
F	3.940		5.030		4.670		6.480					
Prob >F	0.000		0.000		0.000		0.000					
R^2^	0.386		0.413		0.386		0.413		0.386		0.413	
Adj, R^2^	0.227		0.260		0.227		0.260		0.227		0.260	

Note: Dependent variable is WTP for local; Standard errors in parentheses; p-values in brackets. CI refers to 95% confidence interval.

***, **, * indicate significance at the 1%, 5%, and 10%-level, respectively. *sqrt(n)* is used as weight.

The estimated constant which serves as a proxy for the “true” WTP for local, after correcting for publication bias is our main parameter of interest. The results in Table 3 indicate the presence of a significant WTP for local effect. That is, the constant estimate is significant in all of our models, implying that, after controlling for publication bias, the weighted average of WTP for local across the included studies ranges between $1.696/lb and $2.076/lb.

Table 4 presents the results of the full MRA models that take into account methodological differences across the studies included in our analysis. Model diagnosis shows that all models are overall significant based on the F-test. S4 Table reports that none of the variance inflation factors (VIFs) values exceeds the critical level of 10. Moreover, the correlation matrix reported in S2 Table does not point to high correlations among the set of independent variables. This indicates that a severe degree of multicollinearity among the set of included explanatory variables is not present. To assess the remaining degree of heteroscedasticity after employing a weighted estimation technique, we calculate Breusch-Pagan (BP) test statistics for the final models. Results show that in none of the cases the null hypothesis of homoscedasticity is rejected (Chi^2^ = 11.32, p = 0.660, df = 14 for the specification that uses *sqrt(n)* as a precision measure; Chi^2^ = 11.78, p = 0.624, df = 14 for the specification using the number of participants as precision measure).

Similar to the simple model (Table 3), in our interpretation we focus on the main specification based on WLS with cluster robust standard errors. These results are presented in columns (3) and (4), while the remaining specifications shall serve as robustness tests. The results confirm the presence of publication bias as the coefficients for *sqrt(n)* and *n* remain significant and negative after the inclusion of relevant study design covariates. Moreover, the estimated constant terms are significant and positive. This indicates that after controlling for publication bias and variation in the WTP due to methodological and other study-specific characteristics, a premium for local attribute remains present. Note that the constant reflects the “true” empirical WTP for local beyond publication bias if all study design covariates are equal to zero [89]. Hence, one should be careful when interpreting the constant parameter because the results suggest that several covariates included in the model have a significant effect. We discuss this in more detail below.

Several covariates related to the study design turn out to be significant. We find that the *Year of study estimate* is significant and negative, implying that consumers’ WTP for local food has decreased over time, perhaps because it became more widely available as even supermarkets are now increasingly offering locally sourced products [90]. On the other hand, the estimated coefficient of *Country of study-US* is significant and positive, indicating that the studies that were carried out in the US reported a significantly higher WTP for local food than non-US studies. Therefore, researchers, farmers and policymakers should be careful when generalizing the results from different countries.

With regards to the products analyzed, we find a significantly higher WTP for local *Animal products* and *Processed products* compared to local *Produce*, the base group of the estimation. This indicates that the price premium for processed and, hence, value-added local food products is higher than for unprocessed alternatives, such as produce [66]. This finding also seems to be consistent with the previous research that considers multiple products in their studies, and reports higher WTP for local animal products compared to local produce [37, 91]. Future studies should take these results into consideration when deciding on the type of a product to utilize for their analysis.

We find no evidence that reported WTP vary depending on the definition of “local” used in the study. This suggests that consumers do not seem to differentiate between the various labels used to convey the fact that the product is produced locally. Labels based on a general definition of “local”, State Grown labels, as well as, labels referring to a specific region yield the same WTP estimates as labels related to marketing programs (the base category of the regression model). The goal of marketing programs is often to increase consumers’ WTP. The fact that these labels result in similar WTP as those related to the other definitions indicates that they might lack awareness and support among consumers [92]. This might be due to the fact that they are not widely promoted and explained.

The significant and positive *Method—choice experiment* variable indicates that employing choice experiments can lead to higher WTP estimates as compared to the application of other experimental techniques, including auctions and contingent valuation. This is in line with Gracia et al. (2012 [59]) and Grebitus et al. (2013a [93]) who find that WTP measures from choice experiments and auctions differ. The coefficient of *Hypothetical* is insignificant, suggesting that there is no significant difference between the WTP obtained by conducting hypothetical and non-hypothetical experiments. This finding is consistent with previous research that shows that hypothetical studies are a good representation of non-hypothetical settings and provide bias free estimates of marginal WTP [67, 68, 69, 70].

Also, we find that the *Number of attributes* used in the study has no effect on WTP for local. Similarly, our results suggest that there is no difference in reported WTP among studies that use store shoppers as compared to participants recruited through a market research company, online-survey database or through mailing and calling (the base category of the regression model). This implies that samples of randomly recruited participants through various channels, including on-line, yield similar results to samples that use “real” shoppers as participants. This is consistent with prior research [94, 95, 96]. However, while we also find no evidence that reported WTP estimates vary significantly over *Age*, *Gender* of the study participants has a significant negative effect on the resulting WTP for local, indicating that female consumers might be more price conscious compared to male consumers. The variable income was excluded from the estimation as it is highly correlated with the Country of Study-US variable leading to severe multicollinearity problems.

To interpret the estimated coefficient of the constant we use model (4) as an example. Setting all categorical variables related to the underlying study design equal to zero, inserting mean values for the variables *participants (n)* and *Gender*, and adding the constant yields a statistically significant value of 1.89. This suggests the presence of a significant WTP for local of 1.89 $/lb after correction for publication bias for the base group (before 2011, non-US, produce, marketing definition of local, no choice experiment or hypothetical, participants recruited outside the shopping locations, and zero additional attributes). Starting from this value we can calculate the WTP for each combination of study design attributes. For example, focusing on the US increases this value by 0.79, while considering studies post 2011 leads to a decrease of 0.81.

Note that the small number of available observations [69] compared to the rather large number of explanatory variables included [14] might point to a problem of model overfitting. Nevertheless, recommendations on model overfitting by Howell (1987 [97]) and Harris (1985 [98]) state that the number of observations should exceed the number of regressors by 50 which is fulfilled in the present case.

Finally, directly taking WTP as reported in the underlying paper might result in biases caused by, e.g., heterogeneity across products or countries that cannot be controlled for by the explanatory variables included in the MRA [99]. Therefore, as additional robustness check, we estimated our models using the percentage of WTP premium over the average price of the product (e.g., [63]) as a dependent variable. The results are presented in Tables A and B in S3 Table, and mainly reflect our original findings. In addition, a funnel plot using the percentage WTP premium also reflects the plot using the direct WTP measure (see S1 Fig).

## Conclusion

The body of research on local food continues to grow, with many articles investigating the premium consumers are willing to pay for local. This literature, however, provides a range of estimates for the local attribute that appears to vary significantly based on, for example, the type of “local” labeling employed [38, 48, 35, 32, 39] or the product category used [15, 26, 22, 17, 11]. Therefore, the objective of this paper is to determine a holistic estimate of the WTP for the local attribute. In order to do so, we utilize an MRA, which is a quantitative method used to evaluate the effect of study-specific characteristics on published empirical results. Collecting all relevant evidence on this topic and utilizing a systematic review methodology, we find that there is a significant mean estimate for products labeled as “local” that ranges between $1.70/lb and $2.08/lb (0.414 and 0.522 when the percentage WTP premium is used). As such, this research contributes to the broad literature that studies consumer demand for local food by deriving a proxy for “true” WTP for local.

In addition, we detect publication bias in the literature reviewed because we identify a significant relationship between WTP for “local” estimates and their precision. This suggests that there might be a tendency to select a specific combination of estimates and precision that leads to statistical significance, implying that significant estimates are more likely to be selected for publication.

Using our analysis, we also examine how major methodological characteristics of the studies affect the WTP estimates. For example, our results indicate that the specific labelling used by a study to convey the “local” attribute does not affect the reported results. This suggests that consumers do not seem to favor any particular definition of local. Therefore, policymakers and farmers should take our findings into consideration when deciding on how to promote local food. For example, investing in marketing program labels seems inefficient because they do not have a significant effect on consumers’ WTP. This might occur due to the lack of awareness of such programs.

Instead of looking to increase the sales through the use of various labels, farmers should consider extending their product lines to include processed and, hence, value-added local items. By extending their product line, farmers will increase the variety of goods available. This, in turn, may improve their profitability because, according to our results, consumers are willing to pay higher premiums for value-added local products compared to local produce. Moreover, it may present farmers with an opportunity to expand their distribution to intermediated channels, since shelf stable items are highly sought there.

Our results also indicate that there is no difference in reported WTP measured by hypothetical as opposed to non-hypothetical experiments. On the other hand, utilizing choice experiments seems to result in higher WTP as compared to other experimental technics, including auctions and contingent valuation. Note that although our findings reveal that the method used (choice experiment vs. other methods) has an impact on the resulting WTP estimate we cannot draw any conclusions on which experimental method is more adequate to use. Therefore, studies that investigate reliability and validity of various research techniques may explore these findings further.

This research is not without limitations. First, the number of studies included is limited by means of the search criteria imposed by our research objective. For example, some articles that do not state “local” explicitly in their title, abstract and/or article keywords, even though they include it in their research design, may have been missed. Therefore, it might be beneficial to repeat our analysis in the future using wider searching criteria. Meanwhile, research on WTP for the local attribute should focus on including all relevant information in the study description, to ensure transparency and allow for in-depth meta-analyses.

Second, while the increasing number of local food articles over time may suggest a rise in interest in local food, it may also indicate that there have been more food or agricultural economics papers published over the years. Future studies should consider looking at the relative rather than the absolute number of articles published. Third, using the year 2011 as a cutoff point to identify whether there is a change in local food preferences over time may be considered shortsighted. Finally, while the majority of studies used in our MRA utilize some variation of the Random Parameters Logit Model for their estimations, five papers employed another type of estimator. However, given the small number, it is not feasible to control for the type of econometric method used. Also, the type of data collection predetermines the type of econometric analysis used. Therefore, including both types of variables would likely lead to multicollinearity between variables related to the econometric method and variables related to the type of data collection. Nevertheless, the estimation method applied might have an effect on the final WTP value reported.

Despite these limitations, the results of this study are valuable because they provide useful information about consumers’ response to labeling and marketing products as local. Our findings can be taken into consideration by future theoretical and empirical research focusing on the WTP for local food. Also, knowing a more precise proxy for the value that consumers place on the local attribute can assist stakeholders from industry, and those involved in policy-making, planning and management, to make better decisions when setting up prices and developing promotional activities.

## Supporting information

S1 Table List of all articles considered. (XLSX)

S2 Table Correlation matrix. (DOCX)

S3 Table Meta regression results. (DOCX)

S4 Table Variance inflation factors. (DOCX)

S1 Fig Funnel plot for WTP (%) for local food estimates. (DOCX)

S1 PRISMA Checklist (DOC)
