# Supplementary material for: The price is right!? A meta-regression analysis on willingness to pay for local food
Source: PLoS One. 2019 May 29;14(5):e0215847. doi: 10.1371/journal.pone.0215847 (PMC6541256; doi:10.1371/journal.pone.0215847)
Supplement: S2 Table — (DOCX) [file pone.0215847.s002.docx]

**S2 Table. Correlation matrix**

|  | WTP % | WTP | sqrt(n) | n | Year of study | Country of study - US | Participants’ origin – shoppers | Local def. – specific region | Local def. – state grown | Local def. – general |
| --- | --- | --- | --- | --- | --- | --- | --- | --- | --- | --- |
| WTP % | 1.000 |  |  |  |  |  |  |  |  |  |
| WTP | 0.499 | 1.000 |  |  |  |  |  |  |  |  |
| sqrt(n) | -0.280 | -0.144 | 1.000 |  |  |  |  |  |  |  |
| n | -0.324 | -0.198 | 0.980 | 1.000 |  |  |  |  |  |  |
| Year of study | 0.239 | 0.143 | -0.228 | -0.289 | 1.000 |  |  |  |  |  |
| Country of study - US | -0.126 | 0.152 | 0.328 | 0.307 | -0.116 | 1.000 |  |  |  |  |
| Participants’ origin – shoppers | 0.058 | 0.053 | -0.324 | -0.362 | 0.036 | -0.181 | 1.000 |  |  |  |
| Local def. – specific region | -0.066 | -0.111 | 0.186 | 0.242 | -0.027 | -0.433 | 0.156 | 1.000 |  |  |
| Local def. – state grown | 0.133 | 0.227 | -0.081 | -0.113 | 0.122 | 0.349 | -0.055 | -0.427 | 1.000 |  |
| Local def. – general | 0.074 | -0.081 | -0.069 | -0.100 | 0.037 | 0.120 | -0.250 | -0.302 | -0.229 | 1.000 |
| Processed products | -0.290 | -0.106 | 0.294 | 0.376 | -0.088 | 0.066 | 0.081 | 0.191 | -0.256 | -0.192 |
| Animal products | 0.298 | 0.199 | -0.184 | -0.250 | 0.351 | -0.258 | -0.171 | 0.081 | 0.053 | -0.079 |
| Method – choice experiment | 0.229 | 0.254 | 0.312 | 0.232 | 0.310 | 0.361 | 0.116 | 0.023 | 0.151 | 0.065 |
| Hypothetical experiment | -0.178 | -0.048 | 0.373 | 0.303 | -0.101 | 0.435 | -0.345 | -0.479 | 0.214 | 0.200 |
| Age | -0.196 | -0.138 | 0.337 | 0.347 | 0.032 | 0.196 | -0.023 | 0.229 | -0.081 | -0.117 |
| Number of attributes | -0.330 | -0.229 | 0.774 | 0.786 | -0.183 | 0.272 | -0.337 | 0.135 | -0.115 | -0.011 |
| Gender | -0.100 | -0.046 | -0.284 | -0.260 | -0.222 | 0.252 | 0.045 | -0.402 | 0.228 | -0.033 |

|  | Processed products | Animal products | Method – choice experiment | Hypothetical experiment | Age | Number of attributes | Gender |
| --- | --- | --- | --- | --- | --- | --- | --- |
| Processed products | 1.000 |  |  |  |  |  |  |
| Animal products | -0.572 | 1.000 |  |  |  |  |  |
| Method – choice experiment | 0.046 | 0.011 | 1.000 |  |  |  |  |
| Hypothetical experiment | 0.072 | -0.099 | 0.221 | 1.000 |  |  |  |
| Age | 0.129 | -0.078 | 0.227 | -0.048 | 1.000 |  |  |
| Number of attributes | 0.405 | -0.167 | 0.385 | 0.384 | 0.215 | 1.000 |  |
| Gender | 0.048 | -0.172 | -0.062 | 0.341 | -0.218 | -0.101 | 1.000 |
