# Supplementary material for: The price is right!? A meta-regression analysis on willingness to pay for local food
Source: PLoS One. 2019 May 29;14(5):e0215847. doi: 10.1371/journal.pone.0215847 (PMC6541256; doi:10.1371/journal.pone.0215847)
Supplement: S3 Table — (DOCX) [file pone.0215847.s003.docx]

**S3 Table A. PET and FAT analysis (using WTP %)**

|  | **WLS with robust SEs** | | | | | | **WLS with cluster robust SEs** | | | | **Wild bootstrap cluster robust SEs** | | | | | |
| --- | --- | --- | --- | --- | --- | --- | --- | --- | --- | --- | --- | --- | --- | --- | --- | --- |
|  | (1) | | | (2) | | | (3) | | (4) | | (5) | | | (6) | | |
|  | Coeff. | | CI | Coeff. | | CI | Coeff. | CI | Coeff. | CI | Coeff. | CI | | Coeff. | | CI |
| Constant | 0.522*** (0.065) | | 0.393; 0.651 | 0.414*** (0.042) | | 0.330; 0.497 | 0.522*** (0.097) | 0.323; 0.721 | 0.414*** (0.062) | 0.287; 0.540 | 0.522*** [0.002] | 0.341; 0.702 | | 0.414*** [0.000] | | 0.296; 0.527 |
| sqrt(n) | -0.009*** (0.002) | | -0.013; -0.005 |  |  | | -0.009*** (0.003) | -0.015; -0.003 |  |  | -0.009*** [0.006] | -0.015; -0.004 | |  |  | |
| n |  |  | | -0.000*** (0.000) | | -0.000; -0.000 |  |  | -0.000*** (0.000) | -0.000; -0.000 |  | |  | -0.000*** [0.002] | | -0.000; -0.000 |
| obs | 73 | | | 73 | | | 73 | | 73 | | 73 | | | 73 | |  |
| F | 24.48 | | | 35.42 | | | 10.50 | | 16.13 | |  |  | |  | |  |
| Prob > F | 0.000 | | | 0.000 | | | 0.003 | | 0.000 | |  |  | |  | |  |
| R^2^ | 0.174 | | | 0.207 | | | 0.174 | | 0.207 | | 0.174 | | | 0.207 | | |
| Adj, R^2^ | 0.163 | | | 0.196 | | | 0.163 | | 0.196 | | 0.163 | | | 0.196 | | |

Note: Dependent variable is WTP for local; Standard errors in parentheses; p-values in brackets. CI refers to 95% confidence interval

***, **, * indicate significance at the 1%, 5%, and 10%-level, respectively. *sqrt(n)* is used as weight.

**S3 Table B. Meta Regression Results (using WTP %)**

|  | WLS with robust SEs | | | | | WLS with cluster robust SEs | | | | Wild bootstrap cluster robust SEs | | | |  |  |  |
| --- | --- | --- | --- | --- | --- | --- | --- | --- | --- | --- | --- | --- | --- | --- | --- | --- |
|  | (1) | | | (2) | | (3) | | (4) | | (5) | | (6) | | | |  |
|  | Coeff. | CI | | Coeff. | CI | Coeff. | CI | Coeff. | CI | Coeff. | CI | Coeff. | CI | |  |  |
| Constant | 1.675*** (0.525) | 0.619; 2.731 | | 1.519*** (0.497) | 0.519; 2.519 | 1.675*** (0.478) | 0.681; 2.669 | 1.519*** (0426) | 0.631; 2.406 | 1.675*** [0.004] | 0.896; 2.460 | 1.519*** [0.002] | 0.859; 2.199 | |  |  |
| sqrt(n) | -0.009 (0.007) | -0.023; 0.004 | |  |  | -0.009 (0.006) | -0.221; 0.195 |  |  | -0.009 [0.350] | -0.021; 0.002 |  |  | | | |
| n |  | |  | -0.000* (0.000) | -0.000; 0.000 |  |  | -0.000* (0.000) | -0.001; 0.000 |  |  | -0.000 [0.198] | -0.000; -0.000 | |  |  |
| Year of study | -0.013 (0.088) | -0.190; 0.164 | | -0.042 (0.088) | -0.219; 0.136 | -0.013 (0.100) | -0.221; 0.195 | -0.042 (0.100) | -0.249; 0.165 | -0.013 [0.904] | -0.175; 0.149 | -0.042 [0.746] | -0.207; 0.117 | |  |  |
| Country of study - US | -0.095 (0.104) | -0.304; 0.115 | | -0.097 (0.100) | -0.229; 0.105 | -0.095 (0.115) | -0.334; 0.145 | -0.097 (0.107) | -0.320; 0.125 | 0.095 [0.604] | -0.266; 0.084 | -0.097 [0.544] | -0.258; 0.070 | |  |  |
| Animal products | 0.158** (0.078) | 0.000; 0.316 | | 0.156** (0.079) | -0.004; 0.316 | 0.158* (0.091) | -0.030; 0.347 | 0.156 (0.096) | -0.043; 0.355 | 0.158 [0.160] | 0.008; 0.320 | 0.156 [0.206] | -0.005; 0.324 | |  |  |
| Processed products | 0.165* (0.094) | -0.024; 0.354 | | 0.185* (0.096) | -0.008; 0.379 | 0.165 (0.146) | -0.140; 0.470 | 0.185 (0.151) | -0.129; 0.500 | 0.165 [0.488] | -0.075; 0.405 | 0.185 [0.456] | -0.062; 0.436 | |  |  |
| Local def. – state grown | 0.146 (0.112) | -0.081; 0.372 | | 0.140 (0.108) | -0.078; 0.358 | 0.146 (0.143) | -0.152; 0.443 | 0.140 (0.137) | -0.145; 0.425 | 0.146 [0.410] | -0.095; 0.378 | 0.140 [0.430] | -0.097; 0.362 | |  |  |
| Local def. – specific region | -0.054 (0.061) | -0.177; 0.069 | | -0.046 (0.061) | -0.169 0.076 | -0.054 (0.069) | -0.196 0.089 | -0.046 (0.068) | -0.188; 0.096 | -0.054 [0.498] | -0.163; 0.056 | -0.046 [0.560] | -0.155; 0.065 | |  |  |
| Local def. – general | 0.010 (0.124) | -0.239; 0.260 | | -0.011 (0.119) | -0.250; 0.227 | 0.010 (0.139) | -0.279; 0.300 | -0.011 (0.127) | -0.275; 0.252 | -0.010 [0.940] | -0.216; 0.236 | -0.011 [0.978] | -0.220; 0.194 | |  |  |
| Method – choice experiment | 0.315*** (0.108) | 0.097; 0.533 | | 0.290*** (0.104) | 0.081; 0.499 | 0.315*** (0.134) | 0.036; 0.594 | 0.290*** (0.120) | 0.040; 0.540 | 0.315* [0.078] | 0.098; 0.542 | 0.290** [0.044] | 0.092; 0.498 | |  |  |
| Hypothetical experiment | -0.006 (0.105) | -0.218; 0.207 | | -0.017 (0.104) | -0.226; 0.192 | -0.006 (0.089) | -0.191; 0.179 | -0.017 (0.086) | -0.196; 0.162 | -0.006 [0.950] | -0.147; 0.144 | -0.017 [0.848] | -0.150; 0.126 | |  |  |
| Participants’ origin – shoppers | -0.123 (0.115) | -0.354; 0.109 | | -0.157 (0.118) | -0.393; 0.080 | -0.123 (0.114) | -0.359; 0.114 | -0.157 (0.110) | -0.386; 0.073 | -0.123 [0.406] | -0.310; 0.072 | -0.157 [0.250] | -0.339; 0.031 | |  |  |
| Number of attributes | -0.037 (0.051) | -0.139; 0.064 | | -0.018 (0.051) | -0.121; 0.085 | -0.037 (0.058) | -0.158; 0.083 | -0.018 (0.058) | -0.138; 0.102 | -0.037 [0.700] | -0.139; 0.056 | -0.018 [0.842] | -0.119; 0.078 | |  |  |
| Age | -0.019** (0.009) | -0.038; -0.000 | | -0.017* (0.009) | -0.036; 0.002 | -0.019* (0.010) | -0.040; 0.002 | -0.017* (0.009) | -0.037; 0.002 | -0.019 [0.214] | -0.036; -0.002 | -0.017 [0.204] | -0.033; -0.001 | |  |  |
| Gender | -0.006* (0.004) | -0.013; 0.001 | | -0.006* (0.003) | -0134; 0.001 | -0.006* (0.003) | -0.012; 0.001 | -0.006** (0.003) | -0.013; -0.000 | -0.006 [0.110] | -0.011; -0.001 | -0.006** [0.002] | -0.011; -0.002 | |  |  |
| obs | 61 | | | 61 | | 61 | | 61 | | 61 | | 61 | | | |  |
| F | 5.79 | | | 6.10 | | 5.79 | | 6.10 | |  | |  | | | |  |
| Prob > F | 0.000 | | | 0.000 | | 0.000 | | 0.000 | |  | |  | | | |  |
| R^2^ | 0.500 | | | 0.516 | | 0.500 | | 0.516 | | 0.500 | | 0.516 | | | |  |
| Adj, R^2^ | 0.348 | | | 0.369 | | 0.348 | | 0.369 | | 0.348 | | 0.369 | | | |  |

Note: Dependent variable is WTP for local; Standard errors in parentheses; p-values in brackets. CI refers to 95% confidence interval.

***, **, * indicate significance at the 1%, 5%, and 10%-level, respectively. *sqrt(n)* is used as weight.
