# Supplementary material for: The price is right!? A meta-regression analysis on willingness to pay for local food
Source: PLoS One. 2019 May 29;14(5):e0215847. doi: 10.1371/journal.pone.0215847 (PMC6541256; doi:10.1371/journal.pone.0215847)
Supplement: S4 Table — (DOCX) [file pone.0215847.s004.docx]

**S4 Table. Variance Inflation factors**

| **Variable** | **VIF** | **VIF** |
| --- | --- | --- |
| sqrt(n) | 5.73 | - |
| n | - | 7.19 |
| Year of study | 1.84 | 1.95 |
| Country of study – US | 1.94 | 1.94 |
| Animal products | 2.17 | 2.17 |
| Processed products | 2.80 | 2.86 |
| Local def. – state grown | 1.90 | 1.90 |
| Local def. – specific region | 2.62 | 2.65 |
| Local def. – general | 2.40 | 2.46 |
| Method – choice experiment | 1.94 | 1.92 |
| Hypothetical experiment | 2.37 | 2.18 |
| Participants’ origin – shoppers | 1.98 | 2.23 |
| Number of attributes | 4.20 | 4.73 |
| Age | 1.31 | 1.35 |
| Gender | 2.31 | 2.13 |
| Mean VIF | 2.54 | 2.69 |
|  |  | |
