# Supplementary material for: The price is right!? A meta-regression analysis on willingness to pay for local food
Source: PLoS One. 2019 May 29;14(5):e0215847. doi: 10.1371/journal.pone.0215847 (PMC6541256; doi:10.1371/journal.pone.0215847)
Supplement: S1 Fig — (DOCX) [file pone.0215847.s005.docx]

Note: “True” values indicated by vertical lines are generated by averaging the 10% and 20% most precisely estimated WTP effects

**S1 Fig.** **Funnel plot for WTP (%) for local food estimates**
